# Long Non-Coding RNA NRON promotes Tumor Proliferation by regulating ALKBH5 and Nanog in Gastric Cancer

**DOI:** 10.7150/jca.60737

**Published:** 2021-09-27

**Authors:** Shuchang Wang, Yangyang Wang, Zizhen Zhang, Chunchao Zhu, Chaojie Wang, Fengrong Yu, Enhao Zhao

**Affiliations:** Department of Gastrointestinal Surgery, Ren Ji Hospital, Shanghai Jiao Tong University School of Medicine, Shanghai, 200127, China.

**Keywords:** Gastric cancer, NRON, ALKBH5, Nanog, m6A

## Abstract

Long non-coding RNAs (lncRNAs) act as tumor suppressors or oncogenes in tumor development and progression. In this study, we explored the expression and biological role of lncRNA NRON in gastric cancer (GC). We observed that lncNRON was upregulated in GC tissues and cell lines, and high lncNRON expression was associated with malignant features and poor prognosis in GC patients. LncNRON was found to promote the proliferation and tumorigenicity of GC cells. Mechanistically, lncNRON exerted its oncogenic functions by binding to the N6-methyladenosine eraser ALKHB5 and mediating Nanog mRNA decay. In conclusion, our results suggest that lncNRON serves as an oncogenic lncRNA in GC and thus may be a promising prognostic factor and potential therapeutic target for GC patients.

## Introduction

Gastric cancer (GC) is a prevalent malignancy around the globe. It is the fifth most common type of malignant tumor and the fourth leading cause of cancer-related mortality worldwide 1-2. GC displays significant global variations, with the highest incidence observed in East Asia 3-4. Early stage GC usually occurs asymptomatically; thus, most GC cases are diagnosed at an advanced stage, and a number of these patients have distant metastasis 5. The prognosis for advanced GC remains unsatisfactory, mainly owing to the high incidence of tumor recurrence and metastasis 6-7. Therefore, a better understanding of the pathogenic mechanisms of GC is needed to develop more efficient diagnosis and treatment strategies for GC patients.

In recent years, we have witnessed a rapid growth in research on RNA modifications, including methylation, acetylation, phosphorylation, and ubiquitylation, all of which occur in various RNAs, such as messenger RNAs (mRNAs), transfer RNAs, and long non-coding RNAs (lncRNAs). N6-methyladenosine (m6A) is the most prevalent modification of eukaryotic RNAs. RNA m6A methylation is reversibly and dynamically mediated by RNA methyltransferases (m6A writers) METTL3 and METTL14 as well as demethylases (m6A erasers) FTO and ALKBH5 8-10. This modification participates in different RNA metabolism processes, resulting in mRNA stability and splicing, translation efficiency, nuclear export, alternative polyadenylation, and microRNA (miRNA) processing. Emerging evidence has shown that RNA m6A modification may play a critical role in the initiation and progression of various cancers. For instance, ALKBH5 maintains tumorigenicity in glioblastoma stem-like cells 11. FTO regulates chemoradiotherapy resistance in cervical squamous cell carcinoma 12. METTL3 controls myeloid differentiation of normal hematopoietic and leukemia cells 13. Nevertheless, the regulatory subunits of m6A RNA erasers and the mechanisms by which m6A affects GC progression remain unclear.

LncRNAs comprise the largest proportion of mammalian non-coding transcripts. LncRNAs are defined as a class of transcripts that are over 200 nucleotides in length without obvious protein-coding potential. LncRNAs have been shown to affect diverse tumor biological processes, including cell proliferation, invasion, metastasis, apoptosis, and chemotherapy resistance 14-15. The molecular mechanisms by which lncRNAs exert their biological functions are diverse and complex; they can serve as signal mediators, molecular decoys, scaffolds or enhancers of transcription, or competing endogenous RNAs 16. However, whether lncRNAs are involved in the m6A modification of RNAs remains unexplored.

In this study, we discovered that the lncRNA NRON is increased in cancer tissues, and patients with high lncNRON levels exhibit more aggressive clinicopathological phenotypes and shorter survival times than patients with low levels. In addition, lncNRON exerts oncogenic functions by binding to the m6A eraser ALKBH5 and inhibiting Nanog mRNA decay. Collectively, our findings revealed the functions of lncNRON and m6A eraser ALKBH5 in GC tumorigenesis, thereby providing potential insights into novel mechanisms and therapeutic strategies for GC.

## Methods

### Patient samples

Tumors and their adjacent gastric tissues were obtained from patients with GC who underwent surgery at Shanghai Renji Hospital between January 2015 and January 2020, with an age range of 31-84 years and ECOG PS 0 or 1. All of the GC tissue specimens were immediately snap‑frozen in liquid nitrogen after surgical resection and then stored at -80°C until total RNA extraction. The eligibility criteria are: (1) All patients underwent radical resection and the postoperative pathology results in all patients verified adenocarcinoma. (2) All patients in this study had integrated clinicopathological data and follow-up information. (3) Patients received neoadjuvant chemotherapy or radiotherapy were excluded. (4) Patients admitted with tumor perforation or hemorrhage for emergency operation were excluded. (5) Patients underwent gastrectomy previously were excluded. (6) Patients suffered other malignant disease within the past 5 years were excluded. (7) Patients died of perioperative complications were excluded.

All procedures performed in this study involving human participants were in accordance with the 1964 Helsinki Declaration ethical standards. The study protocol was approved by the ethics committee of the Shanghai Jiao Tong University School of Medicine, Renji Hospital. Written informed consent was obtained from all participants in this study.

### Cell culture and transfection

The human gastric epithelial cell line GES-1 and the gastric cancer cell lines MKN45, MGC803, and AGS were cultured in RPMI-1640 medium (Basalmedia, China), supplemented with 10% fetal bovine serum (Sigma, China), at 37°C in a humidified atmosphere of 5% CO2. Cell lines were transfected with different siRNAs and plasmids for genetic and functional assays. For cell transfection, 2×10^5^ cells/well were seeded in 6-well plates, and cell transfection was performed using Lipofectamine 2000 (Invitrogen, USA). Full-length NRON was cloned into pcDNA3.1 plasmid. shRNA was inserted into pLKO.1-puro lentiviral plasmid and selected using puromycin. The siRNA and shRNA sequences are depicted in Supplementary Data 1.

### Real-time PCR analysis

Total RNA was extracted from the GC tissues and cell lines using the TRIzol Reagent (Molecular Research Center, USA). For the reverse transcription reaction, 1 μg of total RNA was used with Reverse Transcriptase XL (TAKARA, Japan) in the presence of Oligo(dT) and random primers. QRT-PCR was performed on the QuantStudio 5 real-time PCR system (Applied Biosystems; Thermo Fisher Scientific, Inc., Carlsbad, CA, USA) using the SYBR green reaction mix (Applied Biosystems, USA). For all analysis of the qRT-PCRs (except for cytoplasmic and nuclear RNA isolation assay), β-actin was measured as an internal control. The primer sequences used for PCR are provided in Supplementary Data 1.

### Fluorescence *in situ* hybridization (FISH)

Coverslip-grown cell samples were fixed with methanol for 10 min at room temperature and then permeabilized with 0.5% Triton X-100 for 5 min at 4°C. Then, the cells were incubated with the NRON probe overnight at 37°C. After the samples were washed with the SSC solution, the cell nuclei were stained with DAPI. Images were obtained with a Nikon Eclipse Ti (Nikon, Kanagawa, Japan). The probe mix was designed and purchased from Ribobio (Guangzhou, China).

### Cell proliferation and colony formation assays

MKN-45 and MGC-803 cells were transfected for 48 h and seeded onto 96-well plates (2000 cells/per well) for the CCK assays and also onto 24-well dishes (250 cells/per well). The cell viability was measured daily. The cells were treated with 10 μL of the CCK-8 reagent (DOJINDO, Japan) per well for 1 h. Cell proliferation experiments were performed in quintuplicate. The colonies were observed after 1 week of culture, and then, they were fixed with methanol and stained using crystal violet. For cell count assay, 1×10^5^ transfected cells/well were seeded in 12-well plates, the cells number of every well were harvested the count daily for 3 days.

### *In vivo* tumor assays for tumor formation

A total of 2×10^6^ tumor cells were suspended in 200 μL of PBS and injected the right flank of nude mice. The tumor sizes were measured weekly as soon as the tumors were measurable, and the tumor volumes were calculated using the following formula: volume (mm^3^) = width^2^ (mm^2^) × length (mm)/2. After 4 weeks, the mice were sacrificed, and the tumors were harvested. The xenograft tumor tissues were then subjected to immunohistochemistry for Ki-67 and CD31 staining. All experimental animal protocols were approved by the ethics committee of the Shanghai Jiao Tong University School of Medicine, Renji Hospital (Animal Center of School of Medicine, Shanghai Jiao Tong University).

### Western blot analysis

Whole cell protein lysates were denatured by boiling, and the proteins were separated with SDS-PAGE and transferred onto a nitrocellulose membrane (Millipore). After the membranes were blocked with 5% skim milk for 1 h, the membranes were first incubated overnight with the primary antibodies at 4°C and then with the appropriate horseradish peroxidase-conjugated secondary antibody. The antibodies used for Western blot are provided in Supplementary Data S2.

### RNA pull-down assay

Cells (>10^6^) transfected with 4×S1m tagged expression vector were collected and washed. Then, Protease Inhibitor Cocktail (Millipore) 10 μL and RNase Inhibitor 20 μL (TAKARA) was added. Then, 40 μl washed streptavidin beads (Invitrogen, USA) was used to pulldown the protein-RNA interactions incubating on rotator for overnight. The proteins were precipitated and diluted in 50 μl protein lysis buffer. Finally, the retrieved proteins were measured on SDS-PAGE gels for mass spectrometry or Western blot.

### RNA immunoprecipitation (RIP) assay

RIP experiments were performed using the Magna RIP RNA-binding protein immunoprecipitation kit (Millipore, Massachusetts, USA). Briefly, Protein G Dynabeads (Thermo Fisher Scientific, Carlsbad, California, USA) were incubated with the indicated antibodies for 12h at 4°C with gentle rotation. Cells growing in 15 cm-dishes were lysed by lysis buffer containing protease inhibitors and RNase Inhibitor and centrifuged at 10000 g for 10min. The supernatant was incubated with the Protein G Dynabeads pretreated by indicated antibodies at 4°C overnight. The beads were washed by wash buffer and centrifuged for 6 time. After treated with Proteinase K, the co-precipitated RNA was isolated using TRIzol and detected by QRT-PCR.

### Cytoplasmic and nuclear RNA isolation analysis

Cytoplasmic and nuclear fraction of GC cells was extracted using Thermo Fisher BioReagents (Thermo Fisher Scientific) according to the manufacturer's instructions. QRT-PCR analysis was performed using the SYBR green reaction mix (Applied Biosystems, USA). GAPDH was used as the cytoplasmic endogenous control. U6 small nuclear RNA was used as the nuclear endogenous control.

### Luciferase reporter assay

The promoter sequences of the Nanog were constructed into the firefly-tagged pGL3 promoter luciferase vector and then were co-transfected into GC cells with the Renilla control luciferase vector. Additionally, cells were co-transfected with si-NRON or pcDNA3.1-NRON for another 48 hours. Luciferase activities were measured by dual luciferase assay system and results were presented as the ratio of firefly/Renilla.

### MeRIP-qPCR

Methylated RNA Immunoprecipitation was performed as previously described 17. Briefly, an anti-m6A antibody (NEB, USA) or a corresponding control IgG antibody were incubated with Protein G Dynabeads for 12h at 4°C, followed by incubation with the fragmented RNAs in RIP buffer supplemented with RNase inhibitor for 6 h at 4°C. The immunoprecipitated m6A-methylated RNAs were digested with proteinase K, then extracted by TRIzol and determined by qPCR.

### RNA lifetime assay

Transfected cells (2×10^5^) were seeded in 6-well plates. After 48 h, actinomycin D was added to 5 mg/ml at 12h, 6 h, 3 h and 0 h before harvest. Total RNA was extracted using the TRIzol Reagent. Indicated RNA quantities were determined by RT-qPCR.

### Statistical analysis

Each experiment was repeated at least three times. The statistical analysis was performed using the SPSS 22.0 software (IBM, USA). All of the data were expressed as the mean ± standard deviation. Student's *t*-test or one-way ANOVA was used to compare the means of two or three groups. The correlation between LINC00589 expression and the clinicopathological characteristics was calculated by the chi-square test or Fisher's exact test. A Kaplan-Meier survival curve was employed to evaluate the overall survival, and the log-rank test was used to compare differences between curves. A *P* value of < 0.05 was considered significant (*P < 0.05, ** P < 0.01, and *** P < 0.001).

## Results

### LncNRON is overexpressed and correlated with tumor survival in GC patients

**Figure 1:**
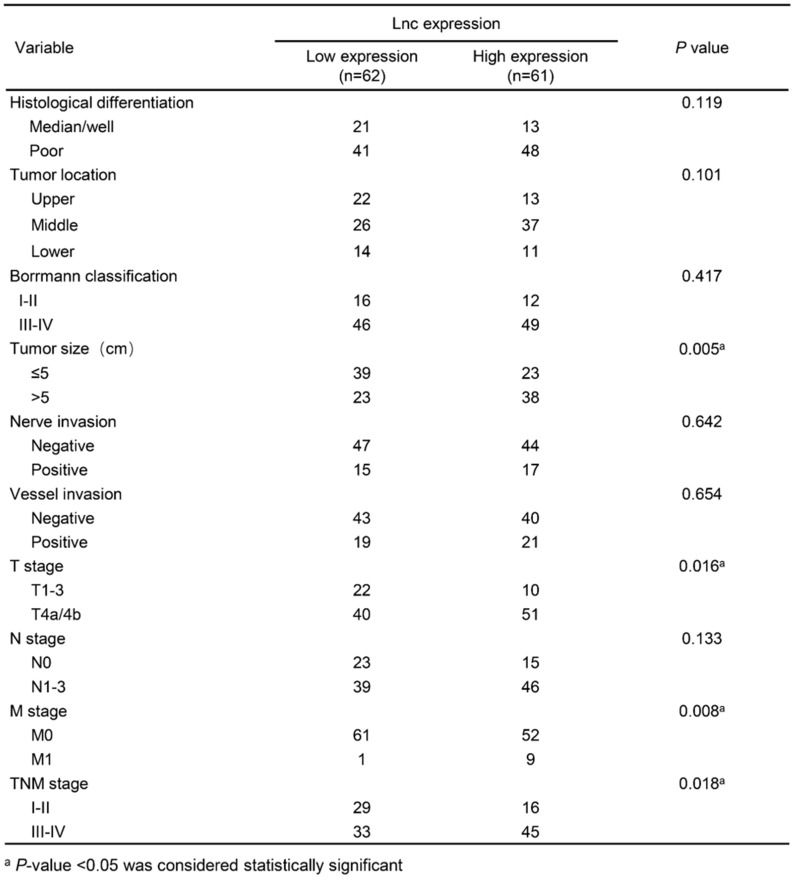

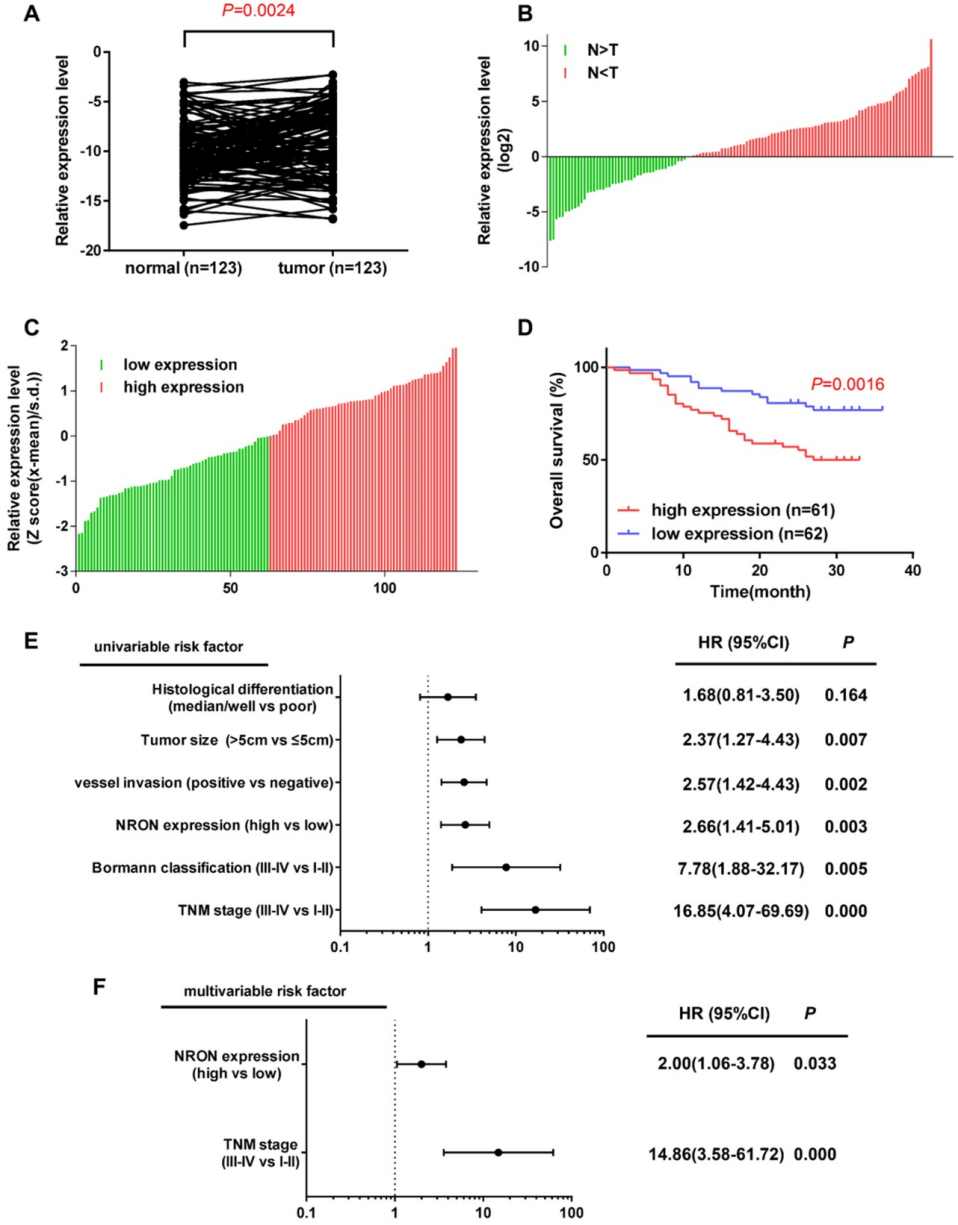
** LncNRON expression is upregulated in GC and is relevant to the prognosis of patients. (A)** Expression of lncNRON in GC and adjacent nontumor gastric tissue specimens. **(B)** The fold change of lncNRON expression in GC compared to nontumor gastric tissue specimens. **(C)** The 123 GC patients were divided into a high lncNRON group (n = 62) and a low lncNRON group (n = 61) according to their median expression levels. **(D)** Kaplan-Meier analysis of the OS of the 123 GC patients based on lncNRON expression. **(E and F)** Univariate analysis (E) and multivariate analyses (F) were performed. All of the bars correspond to 95% confidence intervals.

In this study, we detected the expression level of lncNRON in 123 paired tissues and their corresponding adjacent normal tissues by real-time PCR. The results demonstrated that the expression of lncNRON was elevated in GC tissues compared to that in the corresponding adjacent normal tissues (*P* = 0.0024) (Figure 1A). Among the 123 patients, 63.41% (78/123) showed increased expression of lncNRON (Figure 1B). In addition, the 123 GC patients were divided into a high lncNRON group (n = 61) and a low lncNRON group (n = 62) according to their median expression levels (Figure 1C). Kaplan-Meier analysis using the log-rank test indicated that higher expression of lncNRON was associated with shorter overall survival (OS) (*P* = 0.0016) (Figure 1D). We also evaluated the correlation between lncNRON and clinicopathological characteristics. Statistical analysis showed that lncNRON expression was positively correlated with tumor size, tumor stage, distant metastasis, and tumor-node-metastasis (TNM) stage (Table 1). Univariate (Figure 1E) and multivariate (Figure 1F) regression analyses suggested that lncNRON and TNM stage act as independent predictors of GC prognosis. Collectively, these findings revealed that lncNRON was upregulated in GC tissues and cells, suggesting its potential as a promising prognostic and diagnostic indicator.

### LncNRON promotes GC cell proliferation *in vitro* and *in vivo*

**Figure 2:**
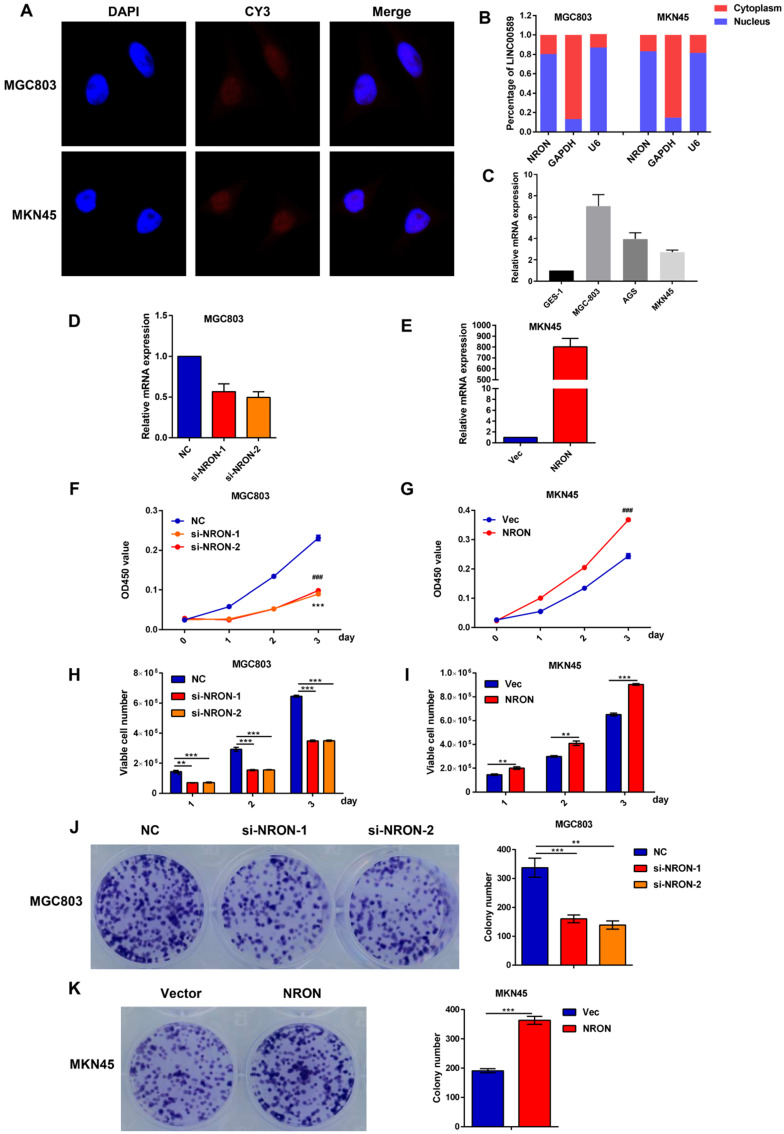
** LncNRON promotes GC cell proliferation and colony formation *in vitro*. (A)** The subcellular location of lncNRON in MGC803 and MKN45 cells was determined using the FISH assay with confocal microscopy. **(B)** The relative distribution of lncNRON in GC cells was detected by cellular fractionation analysis. U6 served as the nuclear marker, and GAPDH served as the cytoplasmic marker. **(C)** LncNRON expression in human GC cell lines (GES, MGC803, AGS, and MKN45). **(D)** The inhibitory efficiency of different siNRON variants was verified by real-time PCR. **(E)** The activation efficiency of lncNRON by pcDNA3.1. **(F and G)** Cell proliferation was determined using the CCK-8 assay after transfection with siRNAs (si1 and si2) or pcDNA3.1-NRON for 24 h.** (H and I)** The cell count assay was performed after transfection with siRNAs (si1 and si2) or pcDNA3.1-NRON. **(J and K)** The colony formation assay was performed in cells that had been transfected for 1 week. Representative images (left) and colony numbers (right) are shown.

We next analyzed the biological roles of lncNRON in GC cells. Fluorescence *in situ* hybridization (FISH) and cellular fractionation analysis demonstrated that lncNRON was mainly located in the nuclei of MGC803 and MKN45 cells (Figure 2A and B). We then detected the NRON expression levels in three human GC cell lines (MGC-803, AGS, and MKN45) and an immortalized human gastric epithelial mucosa cell line (GES-1). The results verified that lncNRON expression was higher in GC cells than in control cells (Figure 2C).

We transfected two small interfering RNAs (siRNAs) and expression plasmids into the MGC803 and MKN45 cell lines to evaluate the effects of lncNRON on cellular behaviors. Real-time PCR was performed to detect the transfection efficiency (Figure 2D and E). To clarify the functional effects of lncNRON expression on cell proliferation, Cell Counting Kit-8 (CCK‑8), cell count, and cell colony formation assays were performed. As shown by the CCK assay and cell count (Figure 2F and G), lncNRON knockdown significantly inhibited GC cell proliferation in the MGC‑803 cell line. Moreover, the colony formation activity of MGC‑803 cells was also impaired by lncNRON downregulation (Figure 2H and I). In contrast, overexpression of lncNRON promote the proliferation and colony formation of MKN45 cells (Figure 2J and K).

**Figure 3:**
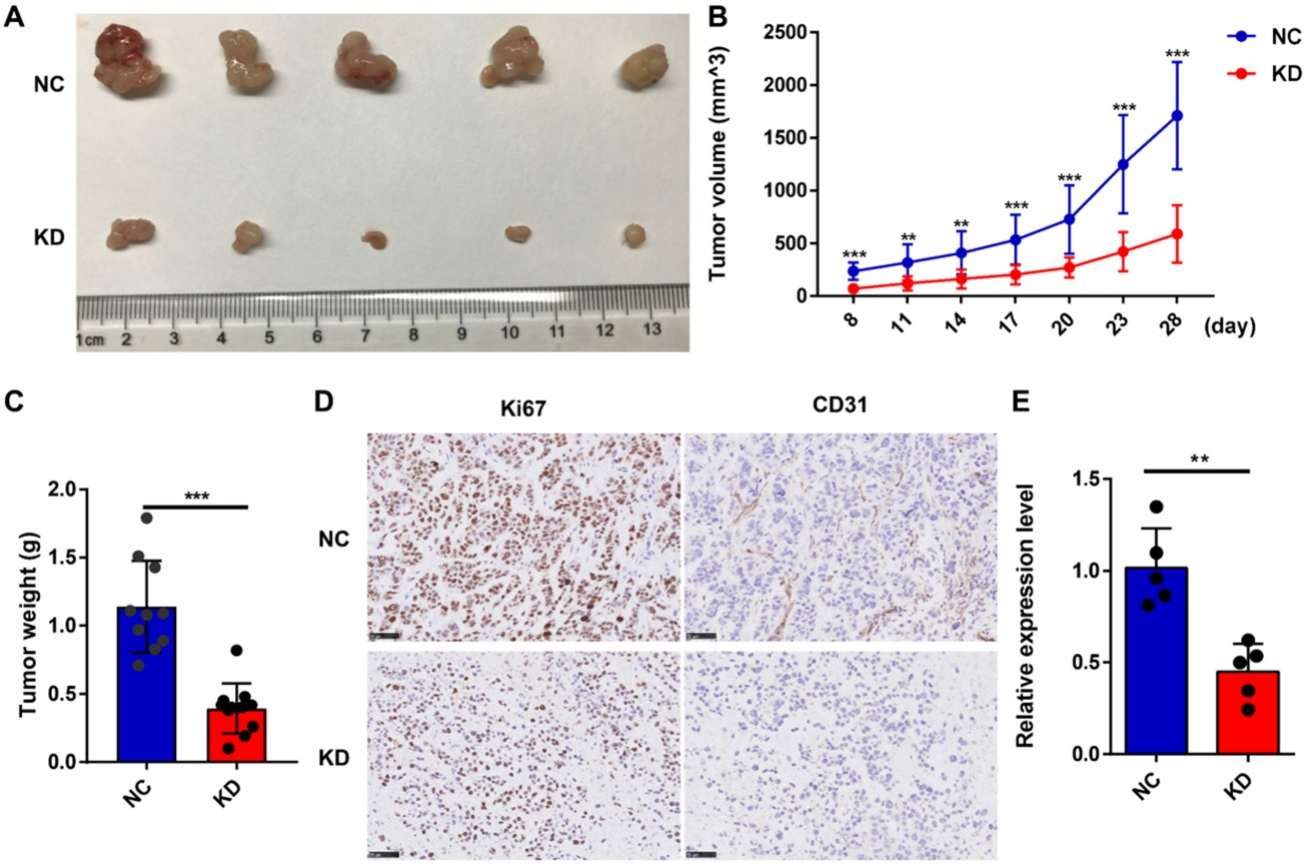
** LncNRON knockdown inhibits the growth of GC cells *in vivo*. (A)** Representative images of nude mice that were subcutaneously injected with transfected GC cells. **(B)** Tumor volumes in the xenograft mouse model were detected weekly. The formula used for the tumor volume calculation was *ab*^2^/2. **(C)** Tumor weight was measured post-sacrifice.** (D)** Representative immunohistochemical images showing the intensity of Ki-67 and CD31 expression of the two groups. **(E)** The expression level of lncNRON in the tumor tissues was evaluated by real-time PCR.

To confirm the effect of lncNRON on GC tumorigenesis *in vivo*, we subcutaneously injected MGC803 cells transfected with short hairpin (sh)-lncNRON or control shRNA into the right flank of nude mice. We found that tumor growth was delayed in the group with lncNRON downregulation (Figure 3A). After 4 weeks, the tumors were harvested, and the tumor volumes and weights for the sh-lncNRON group were considerably impaired (Figure 3B and C). In addition, the expression levels of both Ki-67 and CD31 were significantly inhibited in the sh-lncNRON group compared to those in the control group (Figure 3D). As expected, the lncNRON expression level was remarkably reduced in the tumors derived from the sh-lncNRON group (Figure 3E).

Taken together, these data suggest that lncNRON may exert oncogenic functions and promote the proliferation of GC cells *in vitro* as well as gastric tumor growth *in vivo*.

### LncNRON is associated with Nanog and inhibits its degradation

**Figure 4:**
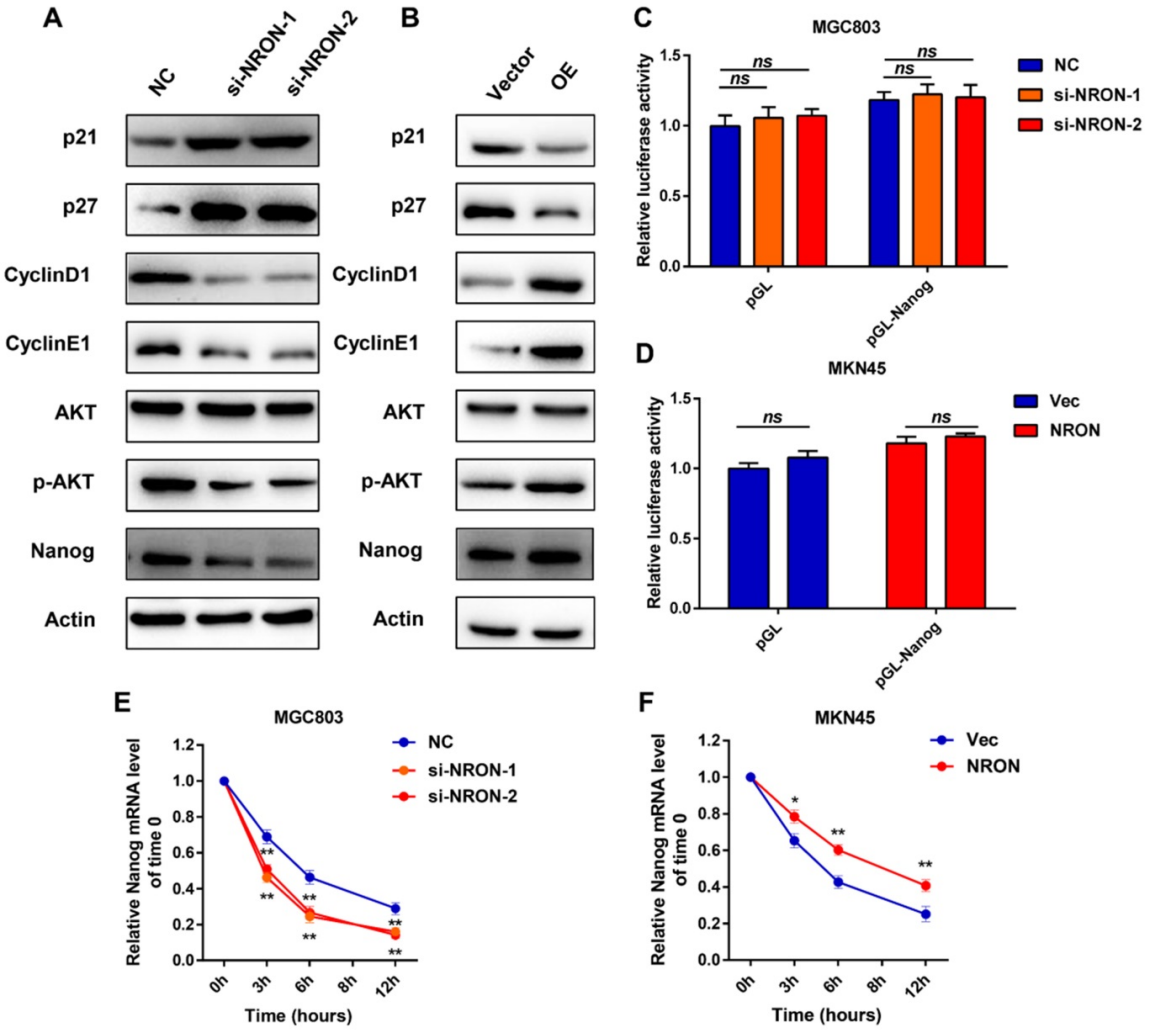
** LncNRON is associated with Nanog and delays its degradation. (A and B)** The western blots of indicated proteins in MGC-803 cells transfected with siNRON (A) and MKN45 cells transfected with pcDNA3.1-NRON (B). **(C and D)** The luciferase activity of the Nanog promoter was detected in lncNRON knockdown (C) or lncNRON overexpression cells (D). **(E and F)** The half-life of Nanog mRNA was detected in lncNRON knockdown (E) or lncNRON overexpression cells (F).

To investigate the mechanism by which NRON activates GC progression, we examined several cell cycles, and markers were detected in the GC cells. The MGC803 cell line was selected for further experiments as it displayed moderate endogenous expression levels of lncNRON. Western blot assays showed that lncNRON expression was negatively correlated with the protein expression of p21 and p27 but positively correlated with the protein expression of cyclin E1 and cyclin D1 in GC cells, suggesting that lncNRON promotes GC cell proliferation by mediating the expression of cell cycle markers (Figure 4A). Moreover, we observed high phosphorylation levels of protein kinase B (Akt) but no change in the total Akt protein amount in GC cells treated with ectopic lncNRON expression compared to control cells. In addition, we observed the upregulation of Nanog with lncNRON overexpression. In contrast, p21, p27, cyclin D1, cyclin E1, phospho-Akt, and Nanog expression levels in GC cells were analysed treated with lncNRON siRNA (Figure 4B). Next, we investigated the regulatory relationship between lncNRON and Nanog, as it is well established that transcription factors play a crucial role in the acquisition of self-renewal properties. A luciferase reporter plasmid containing the Nanog promoter region was constructed and transfected into GC cells. Notably, neither knockdown nor overexpression of lncNRON altered the luciferase activity of the Nanog promoter (Figure 4C and D), suggesting that lncNRON regulates Nanog protein levels at the post-transcriptional level. Therefore, we treated GC cells with α-amanitin to block RNA synthesis and detected the half-life of Nanog mRNA. The results indicated that depletion of lncNRON shortened the half-life of Nanog mRNA in MGC-803 cells (Figure 4E). Inversely, overexpression of lncNRON increased the half-life of Nanog mRNA in MKN45 cells (Figure 4F). These data suggest that lncNRON downregulates Nanog expression by mediating the degradation of Nanog mRNA.

### LncNRON recruits ALKBH5 to regulate Nanog demethylation

**Figure 5:**
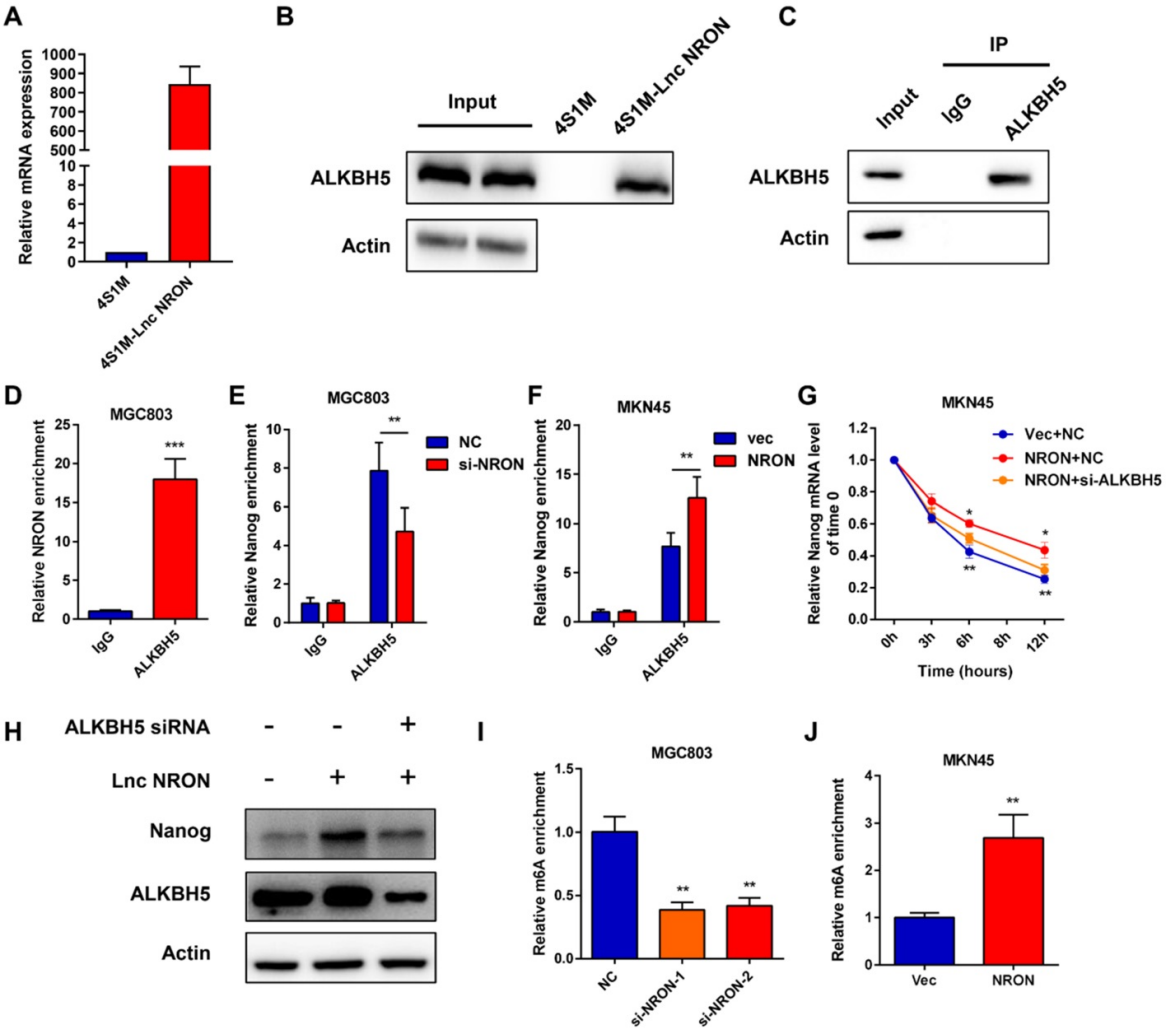
** LncNRON recruits ALKBH5 in order to regulate Nanog demethylation. (A)** 4×S1m tagged lncNRON was constructed as a pull-down probe, and expression of NRON was evaluated. **(B)** RNA pull-down assays were performed to identify the NRON-associated protein via incubation of a 4SA-NRON probe with protein extracts from MGC803 cells. **(C and D)** RIP assays were performed using the ALKBH5 antibody to illustrate the association of NRON with ALKBH5. Immunoblotting analysis (C) and the relative fold change of NRON (D) are shown. **(E and F)** The interaction of Nanog RNA with ALKBH5 was verified after lncNRON knockdown (E) or overexpression (F) by RIP assay. **(G)** The half-life of Nanog mRNA was detected in si-ALKBH5 transfected NRON overexpressioned MKN45 cells.** (H)** Depletion of ALKBH5 expression reversed lncNRON-induced Nanog overexpression.** (I and J)** The m^6^A methylation of Nanog mRNA was detected in lncNRON overexpression (I) or lncNRON knockdown cells (J).

LncRNAs can exert their functions via RNA-protein interactions to modulate target genes. Therefore, we used RNA pull-down assays followed by mass spectrometry to identify the NRON binding protein. Among the top listed potential proteins, we focused on ALKBH5, a well-known m6A demethylase (Supplementary Data 3). Repeated RNA pull-down assays revealed an obvious interaction between NRON and ALKBH5 (Figure 5A and B). The interactions between NRON and ALKBH5 were further confirmed by RNA immunoprecipitation (RIP) assays (Figure 5C and D). It has been well established that ALKBH5 tightly regulates Nanog via m6A demethylase 18-19. To understand the role of lncNRON in the ALKBH5/Nanog axis, we performed an ALKBH5 RIP assay. We observed that overexpression of lncNRON induced the binding of ALKBH5 and Nanog mRNA (Figure 5E). Reciprocally, this interaction was attenuated by lncNRON silencing (Figure 5F). To explore whether lncNRON was involved in ALKBH5-mediated Nanog upregulation, ALKBH5 was downregulated in lncNRON-elevated GC cells. The results showed that knockdown of ALKBH5 remarkably restored the half-life of Nanog with lncNRON overexpression (Figure 5G). Additionally, although lncNRON overexpression had no effect on the protein levels of ALKBH5, ALKBH5 knockdown inhibited lncNRON-stimulated Nanog expression (Figure 5H). Recently, modifications have been found to affect lncRNA-mediated RNA stability. Therefore, we explored the relevance of m6A modification to the lncNRON-mediated regulation of Nanog mRNA degradation. We carried out a methylated RNA RIP assay and observed that knockdown of lncNRON markedly elevated the m6A methylation of Nanog mRNA in MGC-803 cells (Figure 5I), whereas ectopic expression of lncNRON impaired the m6A levels of Nanog mRNA in MKN45 cells (Figure 5J). In conclusion, our findings suggest that lncNRON regulates m6A modification of Nanog mRNA via interactions with ALKBH5.

### ALKBH5 is involved in the process of lncNRON-mediated GC cell proliferation

**Figure 6:**
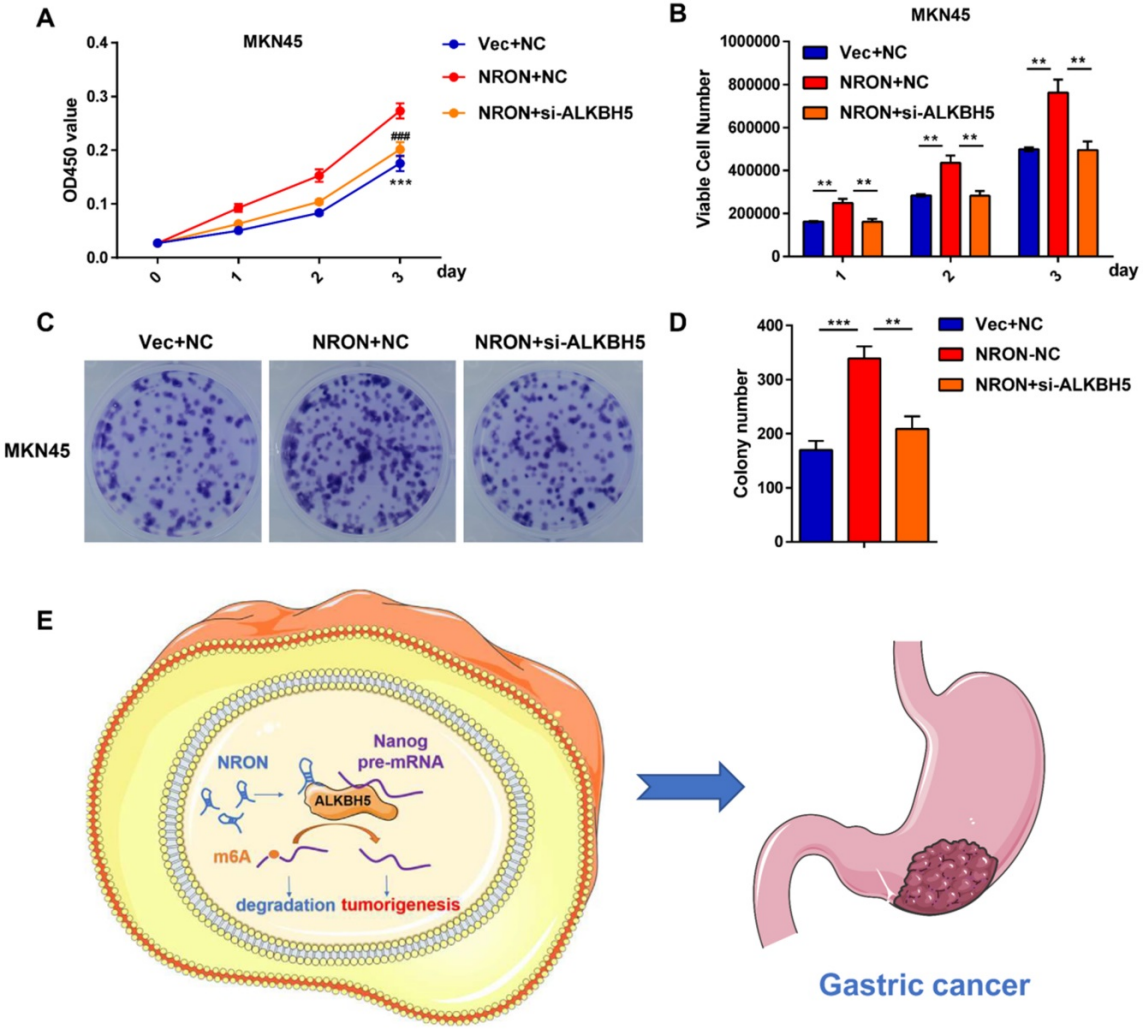
** ALKBH5 is involved in the process of lncNRON-mediated GC cell proliferation. (A)** GC cell proliferation ability was determined using the CCK-8 assay after co-transfection with siNRON and siALKBH5. **(B)** The cell count assay was performed in transfected GC cells. **(C and D)** The colony formation assay was performed in transfected cells. Representative images (C) and colony numbers (D) are shown. **(E)** Schematic model of lncNRON in GC progression. LncNRON promotes GC progression by facilitating Nanog demethylation via ALKBH5 recruitment.

Rescue experiments were performed to identify whether ALKBH5 is involved in the process of lncNRON-mediated GC cell proliferation. CCK-8 (Figure 6A), cell count (Figure 6B), and cell colony formation assays (Figure 6C and D) showed that transiently transfecting Nanog siRNA into lncNRON-overexpressing GC cells significantly inhibited the lncNRON-mediated promotion of cell proliferation. These findings indicate that ALKBH5 promotes GC cell proliferation induced by lncNRON.

## Discussion

Accumulating evidence has revealed that lncRNAs participate in diverse biological processes, suggesting that they may serve as prognostic markers and therapeutic targets for a wide range of tumors. LncNRON was annotated as a suppressor of NFAT by inhibiting nucleocytoplasmic shuttling of NFAT 20-21. However, the latest findings regarding the functions of lncNRON in oncogenesis progression are controversial. LncNRON was found to be reduced in hepatocellular carcinoma (HCC), and high levels of lncNRON attenuate HCC tumorigenesis 22. LncNRON was also downregulated in triple-negative breast cancer (TNBC), and overexpression of lncNRON suppresses lncRNA snaR expression to reduce TNBC cell proliferation 23. However, lncNRON was upregulated in bladder cancer (BC), which promotes the invasion and metastasis of BC cells 24. In this study, we found that lncNRON was upregulated in GC tissues compared to that in adjacent normal tissues. The results showed that lncNRON was upregulated in 63.4% (78/123) of GC patients. The expression of lncNRON was correlated with tumor size, depth of tumor invasion, metastasis stage, and TNM stage. As expected, high lncNRON levels are remarkably associated with unfavorable prognosis in GC cases, suggesting a tumor-promoting function in GC cells. LncNRON also showed oncogenic activity by enhancing GC cell proliferation and colony formation *in vitro*. Additionally, lncNRON knockdown impaired tumor formation in a subcutaneous xenograft model. These results suggest that lncNRON serves as an oncogenic lncRNA in GC cells.

LncRNAs can interact with regulatory proteins, miRNAs, or other cellular factors, which typically serve as pathways to exert their biological functions 25-27. To further elucidate the mechanistic role of lncNRON in GC, we analyzed the potential binding proteins of NRON. Pull-down and RIP experiments showed that ALKBH5 could physically combine with NRON. It is well established that the m6A methylation of NANOG mRNA is tightly regulated by ALKBH5 18-19, which is essential for the acquisition of self-renewal properties in tumors. Our data suggest that lncNRON associations with ALKBH5 could negatively regulate m6A modification of Nanog mRNA, thereby reducing the degradation of Nanog mRNA. Notably, our study will aid in further understanding of the ALKBH5 regulation network and Nanog expression regulation in GC.

The m6A modification is reversible, and its biological effects are regulated by writer, eraser, and reader proteins, which have been identified to be dysregulated in several cancers. Emerging evidence has shown that m6A RNA methylation affects the hallmarks of a variety of cancers. Our previous research revealed that METTL3 mediated m6A modification in human GC progression, wherein it promoted the epithelial-mesenchymal transition process and metastasis 17. However, there are debates regarding the dysregulation of m6A functions in cancer progression. For instance, METTL14 can function as an oncogene in acute myeloid leukemia 28 and simultaneously suppress the metastatic potential of HCC by modulating m6A dependent primary microRNA processing 29. In terms of ALKBH5, it was found that the hypoxic environment in breast cancer cells can induce ALKHB5 expression, which consequently demethylases NANOG mRNA 18. It has also been reported that ALKBH5 is upregulated in glioblastoma cancer stem cells and sustains growth and proliferation by demethylating FOXM1 nascent transcript lncRNA FOXM1-AS 30. However, another study revealed that ALKBH5 can inhibit cell migration and invasion in pancreatic cancer 31. It is known that the same gene can act as a tumor suppressor or oncogene in distinct cancers, contributing to the different interaction partners in diverse cancer cells, which, as a result, execute distinct functions. In this study, we identified lncNRON as a possible additional regulatory subunit of the RNA m6A eraser, which may partially explain the controversial function of ALKBH5 in cancers. Further research is needed to fully verify the molecular mechanism between m6A and lncRNAs.

In summary, a previously annotated lncRNA, lncNRON, exhibits oncogenic roles in the progression of GC. LncNRON, as a possible additional regulatory subunit of the m6A eraser, may strengthen the m6A recognition of RNAs by m6A eraser ALKBH5 to enhance Nanog mRNA stability and expression. Our study uncovered a connection between lncRNAs and the m6A RNA pathway, which may also facilitate further investigation of novel diagnostic and therapeutic strategies for GC patients.

## Supplementary Material

Supplementary tables.

## Figures and Tables

**Table 1:** Associations between lncNRON expression and the clinicopathological features of GC patients

## Abbreviations

lncRNA: long non-coding RNAs
NRON: non-coding repressor of NFAT
GC: gastric cancer
ALKHB5: alkylation repair homolog protein 5
m6A: N6-methyladenosine
mRNAs: messenger RNAs
METTL3: methyltransferase-like 3
METTL14: methyltransferase-like 14
FTO: obesity-associated protein
Akt: protein kinase B
NFAT: T-cell nuclear factor
TNBC: triple-negative breast cancer
BC: bladder cancer
FOXM1: Forkhead box M1
ECOG PS: Eastern Cooperative Oncology Group performance status
