# Supplementary material for: Long Non-Coding RNA NRON promotes Tumor Proliferation by regulating ALKBH5 and Nanog in Gastric Cancer
Source: J Cancer. 2021 Sep 27;12(22):6861–72. doi: 10.7150/jca.60737 (PMC8518016; doi:10.7150/jca.60737)
Supplement: Supplementary file 1 — Supplementary tables. [file jcav12p6861s1.pdf]

Supplementary Data 1. Sequences for the primers, siRNA and shRNA

**Primer name or Sequences of siRNA, shRNA primers:**	**Sequence (5'→3')**
β-actin-F	CATGTACGTTGCTATCCAGGC
β-actin-R	CTCCTTAATGTCACGCACGAT
GAPDH-F	AATCCCATCACCATCTTCCAG
GAPDH-R	CCTTCTCCATGGTGGTGAAGAC
U6-F	CTCGCTTCGGCAGCACA
U6-R	AACGCTTCACGAATTTGCGT
NRON-F	ACGTTCCTTAATGTACGCCTTTGC
NRON-R	TTGGCCGTGTCCTGAGTCCTT
Nanog 3'UTR-F	ATGCAACCTGAAGACGTGTG
Nanog 3'UTR-R	GAGATTGACTGGATGGGCAT
Nanog-F	CAACTGGCCGAAGAATAGCA
Nanog-R	GCAGGAGAATTTGGCTGGAA
4*S1m	ATGCGGCCGCCGACCAGAATCATGCAAGTGCGTAAGATAGTCGCGGGTCGGCGGCCGCATAT<br>GCGGCCGCCGACCAGAATCATGCAAGTGCGTAAGATAGTCGCGGGTCGGCGGCCGCATATG<br>CGGCCGCCGACCAGAATCATGCAAGTGCGTAAGATAGTCGCGGGTCGGCGGCCGCATATGC<br>GGCCGCCGACCAGAATCATGCAAGTGCGTAAGATAGTCGCGGGTCGGCGGCCGCAT
si-NRON-1:	CCAGGCAATGGGAAGCCATAdTdT
si-NRON-2	GCGTTCATGCTCTTTCCACCdTdT
si-ALKBH5	ACAAGTACTTCTTCGGCGAdTdT
sh-NRON-F	CCGGCCAGGCAATGGGAAGCCATATTCAAGAGATATGGCTTCCCATTGCCTGGTTTTTG
sh-NRON-R	AATTCAAAAACCAGGCAATGGGAAGCCATATCTCTTGAATATGGCTTCCCATTGCCTGGGC

Supplementary Data 2. Antibodies used in this study

Antibody	Company	Catalog number
ki67	Cell Signaling Technology	#9449
CD31	Cell Signaling Technology	#3528
Cyclin D1	proteintech	60186-1-Ig
Cyclin E1	proteintech	11554-1-AP
p21	Santa Cruz	sc-397
p27	Santa Cruz	sc-1641
AKT	Cell Signaling Technology	#4691
p-AKT	Cell Signaling Technology	#4060
Nanog	Cell Signaling Technology	#4903
ALKBH5	Millipore	ABE547
β-actin	MBL	PM053-7

Supplementary Data 3. Associated proteins identified by RNA-Pulldown/MS assay

Protein IDs	Protein names	Gene names	MW(Kda)	Score
Q6P6C2	RNA demethylase ALKBH5	ALKBH5	44.3	69.23
P06733	Alpha-enolase OS	ENO1	47.1	56.56
Q08188	Protein-glutamine gamma-glutamyltransferase E	TGM3	76.6	51.84
P02768	Serum albumin	ALB	45.1	41.91
P62191	26S protease regulatory subunit 4	PSMC1	41.1	31.95
P60842	Eukaryotic initiation factor 4A-I	EIF4A1	46.1	31.95
P31153	S-adenosylmethionine synthase isoform type-2	MAT2A	43.6	31.81
Q92785	Zinc finger protein ubi-d4	DPF2	44.2	21.61
O95218	Zinc finger Ran-binding domain-containing protein 2	ZRANB2	36.3	21.59
P82650	28S ribosomal protein S22, mitochondrial	MRPS22	41.3	11.34
